# Assessment of interactions of efavirenz solid drug nanoparticles with human immunological and haematological systems

**DOI:** 10.1186/s12951-018-0349-y

**Published:** 2018-03-15

**Authors:** Neill J. Liptrott, Marco Giardiello, Tom O. McDonald, Steve P. Rannard, Andrew Owen

**Affiliations:** 1 Department of Molecular and Clinical Pharmacology, Institute of Translational Medicine, The University of Liverpool, 70 Pembroke Place, Block H, First Floor, Liverpool, L69 3GF UK; 2 Department of Chemistry, The University of Liverpool, Liverpool, UK; 3 European Nanomedicine Characterisation Laboratory, Department of Molecular and Clinical Pharmacology, Institute of Translational Medicine, The University of Liverpool, Liverpool, UK

**Keywords:** HIV, Antiretroviral, Biocompatibility, Immunotoxicity, Haematotoxicity

## Abstract

**Background:**

Recent work has developed solid drug nanoparticles (SDNs) of efavirenz that have been demonstrated, preclinically, improved oral bioavailability and the potential to enable up to a 50% dose reduction, and is currently being studied in a healthy volunteer clinical trial. Other SDN formulations are being studied for parenteral administration, either as intramuscular long-acting formulations, or for direct administration intravenously. The interaction of nanoparticles with the immunological and haematological systems can be a major barrier to successful translation but has been understudied for SDN formulations. Here we have conducted a preclinical evaluation of efavirenz SDN to assess their potential interaction with these systems. Platelet aggregation and activation, plasma coagulation, haemolysis, complement activation, T cell functionality and phenotype, monocyte derived macrophage functionality, and NK cell function were assessed in primary healthy volunteer samples treated with either aqueous efavirenz or efavirenz SDN.

**Results:**

Efavirenz SDNs were shown not to interfere with any of the systems studied in terms of immunostimulation nor immunosuppression. Although efavirenz aqueous solution was shown to cause significant haemolysis ex vivo, efavirenz SDNs did not. No other interaction with haematological systems was observed. Efavirenz SDNs have been demonstrated to be immunologically and haematologically inert in the utilised assays.

**Conclusions:**

Taken collectively, along with the recent observation that lopinavir SDN formulations did not impact immunological responses, these data indicate that this type of nanoformulation does not elicit immunological consequences seen with other types of nanomaterial. The methodologies presented here provide a framework for pre-emptive preclinical characterisation of nanoparticle safety.

**Electronic supplementary material:**

The online version of this article (10.1186/s12951-018-0349-y) contains supplementary material, which is available to authorized users.

## Background

Nanomedicine offers the potential for therapeutic benefits across indications, sometimes without the cost of discovering new active pharmaceutical ingredients (APIs). However, the behaviour of certain nanomaterials has been demonstrated to differ considerably from larger bulk material [1]. The production and use of engineered nanomaterials is constantly expanding, but there remain uncertainties surrounding the potential risks posed to human health and the environment.

The size of nanomaterials influences their toxicity [1] and has been consistently demonstrated for nanomaterials encountered in workplace environments (e.g. carbon black, polystyrene, titanium dioxide and silver) [2–5]. The physicochemical characteristics of nanomaterials such as charge, surface area, solubility, surface chemistry and shape show a large degree of heterogeneity amongst materials both developed and in development. Immune stimulation, immune suppression and immune modulation have all been reported for nanomaterials [6, 7] partly as a result of the formation of a protein corona made up of serum proteins [8–13]. Opsonisation of nanomaterials by serum proteins such as immunoglobulins and complement proteins results in rapid uptake into cells such as macrophages and dendritic cells [10, 14]. Interactions with serum proteins may render nanomaterials antigenic and it has been suggested that functionalisation (e.g. with growth factors, receptors) may induce neutralising antibodies that also recognise the body’s own molecules with implications similar to those for biotechnology-derived therapeutics [15, 16]. Indeed, anti-nanoparticle antibody formation in response to C_60_ fullerene nanomaterials has been reported [17, 18]. Some studies suggest that nanoparticles can also exacerbate allergic reactions [19]. Immune cells recognise nanoparticles based on their surface properties and core composition and mount inflammatory responses but many of the molecular events are poorly understood [20].

In general, positively charged nanomaterials are more likely to induce inflammatory reactions than negatively charged or neutral materials. For example, positively charged 4.5 amine terminated polyaminoamine (PAMAM) dendrimers do not cause human leukocytes to secrete cytokines [7] but some charged liposomes induce secretion of cytokines such as IL-2 and IFNγ [21]. Several studies have addressed the influence of nanoparticles on Th1 and Th2 responses. Large (> 1 μm) particles have been shown to induce Th1 responses, whereas smaller ones (< 500 nm) are associated with Th2 response [22]. In contrast, some engineered nanoparticles such as 500 nm poly(lactic-*co*-glycolic) acid (PLGA) [23, 24], 270 nm PLGA [25], 80 nm and 100 nm nanoemulsions [26, 27] and 123 nm dendrosomes [28] induce a Th1 response. Other engineered particles (e.g. 5 nm generation-5 PAMAM dendrimers) do not cause inflammatory reactions in vivo, but weakly induce Th2 cytokine production and enhance immunoglobulin production [29]. There are also reports of nanomaterials suppressing immune responses—generation 3.5 PAMAM dendrimers conjugated to glucosamine strongly inhibit induction of inflammatory cytokines in macrophages and dendritic cells exposed to endotoxin [30], and amino-terminated generation-5 PAMAM dendrimers suppress inflammatory cytokine secretion in vitro and in vivo [31]. Conversely, unopsonised silica nanoparticles have been shown to stimulate macrophages to produce inflammatory proteins [32]. Natural killer (NK) cells are an integral part of the innate immune system but also serve to bridge the adaptive immune system [33, 34]. As natural killer cells play a vital role in anti-tumour [35] and anti-viral immunity [36], perturbation of their function may exacerbate existing conditions. Previous work has shown that multi-walled carbon nanotubes suppress NK function in vivo [37].

In addition to interactions with immunological systems there are numerous reports of nanomaterials interacting with components of the blood coagulation system. This has been eloquently reviewed in a recent review paper from the National Cancer Institute’s Nanotechnology Characterisation Laboratory [38]. Oral and intravenous administration of generation-4 and generation-7 PAMAM dendrimers resulted in disseminated intravascular coagulopathy (DIC) in mice [39]. With respect to platelet aggregation, cationic polystyrene latex nanoparticles induced platelet activation and aggregation through cellular membrane perturbation whilst their anionic counterparts activated platelets and induced their aggregation through the classical pathway involving the upregulation of adhesion receptors [40]. Activation of complement has also been demonstrated for perfluorocarbon (PFC)-based emulsions [41]. In addition to activation of these pathways, damage to erythrocytes has been reported for silver nanomaterials where nano-sized particles were found to be significantly more haemolytic than micron-sized particles [42].

Currently, commercial nanomedicines are synthesised as nanocarriers or solid drug nanoparticles (SDNs). Nanocarriers are predominantly parenteral options, targeting therapies to specific cells or tissues, but for chronic diseases daily parenteral administration has consistently failed to meet patient needs, and drug delivery vehicles add risk. SDNs overcome some these problems, providing oral administration formats and more recently, opportunities for long-acting parenteral administration. Our new emulsion-templated freeze-drying technique widens the range of drugs that can be formulated into SDNs to include hydrolytically sensitive APIs [43]. The overwhelming majority of the studies conducted to date have focused upon nanomaterials with a lipid-, polymer-or inorganic composition. There is a current paucity of knowledge regarding whether solid drug nanoparticles (SDNs) elicit such responses, leading to issues with biocompatibility, despite this nanotechnology being the most clinically and commercially successful approach to date. We recently reported that lopinavir SDNs were relatively inert in ex vivo analyses of immunological responses, but there is a need to understand whether this applies across SDNs made from different therapeutic agents [44]. Therefore, we investigated a series of immune-activation/suppression criteria to assess whether the efavirenz SDNs trigger or interfere with immune responses, and to determine their blood contact properties. In order to determine the impact of nanoformulation on immunological effects, all incubations were conducted with matched drug concentrations of efavirenz between aqueous and nanoformulated drug.

## Methods

### Preparation, and analysis, of emulsion-templated freeze-dried monoliths containing efavirenz nanoparticles

Samples are prepared using a 70 mg/mL stock solution of Efavirenz (EFV) in chloroform, a 22.5 mg/mL of poly(vinyl alcohol) (MW = 9500 g/mol, PVA) in water and a 22.5 mg/mL stock solution of α-tocopherol polyethylene glycol succinate (TPGS) in water. Stock solutions are added in the following proportion; 100 µL EFV; 90 µL PVA, 45 µL TPGS and 265 µL of water, therefore solid mass is 10 mg with the ratio; 70% EFV, 20% PVA and 10% TPGS in a 1:4 oil to water (O/W) mix. Where needed, radiolabeled EFV SDNs were prepared by incorporation of ^14^C-labeled EFV (0.1 µCi ^14^C) into the chloroform internal phase of the emulsion during the stock solution preparation. The mixtures are the emulsified using a Covaris S2x for 30 s with a duty cycle of 20, an intensity of 10 and 500 cycles/burst in frequency sweeping mode. After which, the samples were immediately cryogenically frozen and lyophilized using a Virtis benchtop K freeze-drier for 48 h to produce off white dry porous monolith products. Samples were then sealed in individual vials until analysis. In order to determine the dispersed SDN particle characteristics, samples were dispersed by addition of 3.5 mL of water (therefore 1 mg/mL with respect to EFV content). Z-average diameter (*D*_*z*_), Zeta potential (ζ) and polydispersity index (PdI) were determined by dynamic light scattering at a temperature of 25 °C using a Malvern Zetasizer Nano ZS equipped with a 4 mW He–Ne, 633 nm laser and using plastic disposable cuvettes. Malvern Zetasizer software version 6.20 was used for data analysis. Zeta potential measurements were also carried out at 1 mg/mL, 25 °C, and an initial pH of 6.5, using disposable capillary zeta cells. Size, zeta potential and polydispersity measurements were obtained as an average of three individual measurements and were obtained using the instrument’s automatic optimisation settings.

### Assessment of endotoxin concentration in sample material by limulus amoebocyte lysate (LAL) analysis (turbidimetric)

Standard lipopolysaccharide (LPS) from *E. coli* was reconstituted to a final concentration of 1000 EU/mL in pyrogen-free LAL water. Further dilutions were then made in pyrogen-free LAL water to produce a standard curve of 1, 0.1, 0.01 and 0.001 EU/mL . LAL was reconstituted in Glucashield buffer to prevent possible interference by β-glucans that may be present in sample materials. Negative control consisted of pyrogen-free LAL water only and positive control 0.05 EU/mL LPS. Samples of aqueous efavirenz and SDNs were prepared in pyrogen-free LAL water at concentrations of 4 and 40 µg/mL. Inhibition/enhancement (IEC) controls consisted of test samples containing 0.05 EU/mL LPS. Reactions consisted of standard, sample or control with the addition of LAL (50 µL). Samples were then analysed using a Pyros Kinetic Flex reader (American Associates of Cape Cod). Results from each individual assay run were not considered valid unless the precision and accuracy of the standard curve (r^2^ ≥ 0.980) and quality control were within 25%, and the inhibition/enhancement control exhibited 50–200% spike recovery.

### Assessment of endotoxin concentration in sample material by limulus amoebocyte lysate (LAL) analysis (gel-clot)

Samples were prepared as for turbidimetric analysis with the exception of standard curve samples which were prepared as 0.25λ to 2λ (λ is the sensitivity provided for each lot of the lysate by the manufacturer). IEC were prepared in 0.25λ to 2λ samples to assess interference with assay. Samples were incubated for 1 h at 37 °C in an unstirred water bath. Following incubation sample tubes were inverted to assess the formation of a clot.

### Determination of possible microbial contamination in sample materials

To determine if microbial contamination was present in sample materials 50 μL (1 mg/mL) of material was streaked onto LB agar plates and incubated in a humidified incubator at 37 °C for 48 h. *E. coli* stock solution (10 CFU/mL) was used as a positive control for microbial growth. Following incubation, plates were visually inspected for signs of microbial growth and recorded digitally.

### Determination of possible mycoplasma contamination in sample materials

H460 cells were treated with sample materials (4 μg/mL) and passaged every 48 h in RPMI-1640 media containing 10% FBS. At each passage a sample of culture supernatant fraction was retained for mycoplasma analysis. After 18 passages, the first and last passage samples were analysed for the presence of mycoplasma using endpoint PCR containing specific probe sequences as part of a commercially available mycoplasma detection kit (Venor GeM Mycoplasma detection Kit [45]).

### Impact of efavirenz solid drug nanoparticles on platelet aggregation

Platelet rich plasma (PRP) was prepared from healthy volunteer whole blood in sodium citrate by centrifugation at 250×*g* for 8 min. Platelet poor plasma (PPP) was prepared by centrifugation of whole blood at 2500×*g* for 10 min. PRP was treated with either 4 or 40 µg/mL of sample materials (EFV dissolved in DMSO, 0.5% DMSO used for subsequent assays) for 15 min at 37 °C. PPP was used as a background control and also treated with the same concentrations of sample materials. Samples were analysed using a Chrono-log aggregometer, gain was set to 0.005 and optical baseline established using PPP controls. Platelet aggregation (turbidity) and ATP release (luminescence) were recorded as area under the curve for treated and untreated samples. Collagen (1 µg/mL) was used as a positive control for platelet aggregation and materials were also tested in the presence of collagen to ensure inhibition of aggregation did not occur.

### Assessment of plasma coagulation in the presence of nanoparticles

Human blood from three donors was collected by venepuncture into tubes anti-coagulated with sodium citrate; blood was used within 1 h of collection. Test plasma was prepared by centrifuging blood at 2500×*g*, at 21 °C, for 10 min with the resultant plasma collected and pooled. Pooled plasma was stable at room temperature for 8 h. Samples (either conventional EFV or EFV SDN) were prepared at 10 × the required final concentration to accommodate dilution when added to test plasma. Final concentrations tested were 40, 4, 0.8 and 0.16 µg/mL. Samples were mixed with test plasma and incubated at 37 °C for 30 min. Each preparation was prepared in triplicate. Lyophilised controls representing normal and abnormal plasma (plasma with coagulation delay) were reconstituted with distilled water (2 mL) and left to equilibrate to room temperature 30 min prior to use. These controls are used as instrumentation controls for the STArt4 coagulometer (Diagnositca Stago). Instrument test parameters (as outlined in Additional file 1: Table S1) were set up for each assay target. Assays are designed to capture interactions of nanoemulsions with components of the three major coagulation pathways; intrinsic pathway (also known as the contact activation pathway, because it is activated by a damaged surface), extrinsic pathway (also known as the tissue factor pathway) and the final common pathway. Activated partial thromboplastin time (APTT) assay is used to assess the intrinsic pathway while the prothrombin time (PT) assay is a measure of the extrinsic pathway. Thrombin time (TT) is an indicator of the functionality of the final common pathway. Cuvettes were placed into A, B, C and D test rows on the coagulometer and one metal ball added into each cuvette (warmed for at least 3 min prior to use). 100 µL of either control or test plasma was added to a cuvette when testing PT or thrombin time and 50 µL when testing APTT with three duplicate cuvettes for each plasma sample. Additionally, for the APTT assays 50 µL of PTT-A was also added. Timer was started for each of the test rows and cuvettes transferred to PIP row 10 s prior to alarm notification. Once incubation time was complete, coagulation reagent (see Additional file 1: Table S1) was added to each cuvette and coagulation time recorded. Percentage coefficient of variation was calculated for each control and test samples according to the following formula:

$$\% {\text{CV}}\, = \,{\text{SD}}/{\text{Mean}} \times 100\% .$$

If %CV was greater than 5% for study samples that sample was reanalysed. Data was expressed as a percentage of the coagulation time recorded for plasma with no SDNs present (plasma only control).

### Determination of possible haemolysis attributed to solid drug nanoparticles

Haemolysis was determined using cyanomethaemoglobin (CMH) reagent and a haemoglobin standard. A standard curve of known haemoglobin concentrations was created (range 0.025–0.8 mg/mL) with low (0.0625 mg/mL), medium (0.125 mg/mL) and high (0.625 mg/mL) quality control samples. Triton X100 was included as a positive control. Sample materials were tested at a range of concentrations (0.16, 0.8, 4 and 40 µg/mL). Whole blood was collected from healthy volunteers in Li-heparin tubes and pooled blood prepared by mixing equal volumes of blood from each donor. An aliquot of pooled whole blood was taken and centrifuged at 800×*g* for 15 min to determine plasma free haemoglobin (PFH). Briefly, 200 µL of calibration standard, quality controls and blanks were added to respective wells of a 96 well plate. Total blood haemoglobin (TBH) (200 µL, prepared by combining 20 µL of pooled whole blood and 5 mL of cyanomethaemoglobin) was added to each well. 100 µL of plasma (for PFH) was added per well. Finally, 100 µL of CMH reagent was added to each well containing samples. Plates were covered with a plate sealer and gently shaken for 1–2 min. Absorbance at 540 nm was read to determine haemoglobin concentration. Remaining pooled whole blood was diluted with Ca^2+^/Mg^2+^ Dulbecco’s phosphate buffered saline (DPBS) to adjust total blood haemoglobin concentration to 10 ± 2 mg/mL (TBHd). In a separate universal tube 100 µL of test sample, blank or positive/negative control was added. Ca^2+^/Mg^2+^ DPBS (700 µL) was then added to each tube and 100 µL of TBHd to each test sample. In parallel 100 µL of Ca^2+^/Mg^2+^ DPBS was added to separate tubes to represent a “no blood” control to evaluate potential interference of the sample materials with the assay. Tubes were covered and mixed gently avoiding vortexing which may damage erythrocytes. Tubes were then placed in an incubator at 37 °C for 3 h (± 15 min) and samples were mixed every 30 min. Following incubation tubes were centrifuged at 800×*g* for 15 min. A fresh set of calibrators and controls were prepared as previously. To a fresh 96 well plate 200 µL of blank reagent, calibrators, and quality controls of TBHd was added to each well. 100 µL of test samples, positive and negative controls were also added to the plate followed by 100 µL of CMH reagent to every well. The plate was covered with a plate sealer and shaken gently on a plate shaker for 1–2 min. Absorbance at 540 nm was measured spectrophotometrically and the %CV and percent difference from theoretical percentage difference from theoretical (PDFT) were calculated. Assays were accepted if %CV and PDFT were within 20%.

### Assessment of possible complement activation by efavirenz solid drug nanoparticles

Blood was collected from healthy volunteers in tubes containing sodium citrate as anticoagulant. Plasma was prepared by centrifugation of blood samples at 2500×*g* for 10 min. Plasma was evaluated visually for haemolysis; plasma deemed to be haemolysed was not used to prepare the plasma pool. Plasma was used for complement testing within 1 h of collection. Pooled plasma was combined with either test material (at concentrations of 0.16, 0.8, 4 and 40 µg/mL) or positive control (cobra venom factor) or negative control (0.9% saline). Plasma was also treated with a generic form of Paclitaxel as a clinically relevant comparison for possible complement activation. Samples were incubated for 30 min at 37 °C. Following incubation, the manufacturers guidelines were then followed for completion of the iC3b ELISA. Optical density of the samples was measured at 405 nm.

### Isolation of peripheral blood mononuclear cells from peripheral blood samples

Healthy volunteer blood was collected from the National Blood Service (Liverpool, UK) or from healthy volunteers within the department. For the latter, healthy volunteer blood was collected via venepuncture under ethics approval from the University Physical Interventions sub-committee (Reference RETH000563). Informed consent was given and accepted by the healthy volunteers for use of whole blood in subsequent assays. All samples were anonymised. Blood was layered over ficoll in a 2:1 ratio and centrifuged at 2000 rpm for 30 min (4 °C). The peripheral blood mononuclear cell (PBMC) interface was then removed and transferred to a fresh universal tube prior to being washed three times in PBS. PBMC were then counted and resuspended to the required density for subsequent experiments.

### Activation of PBMC using CD2, CD3 and CD28 conjugated MACSiBead particles

Prior to use, MACSiBead particles were prepared according to the manufacturers guidelines. MACSiBead particles (2.5 × 10^6^) were transferred to a sterile universal tube with 200 µL of fresh culture media RPMI-1640, 10% FCS) was added and centrifuged (1500 rpm) for 5 min. The supernatant fraction was aspirated and MACSiBeads were resuspended in 100 µL of fresh culture media. PBMC were resuspended in culture media (900 µL) at a density of 5 × 10^6^ cells. PBMC and MACSiBeads were combined and incubated at 37 °C for 24 h. Culture conditions consisted of unactivated PBMC, MACSiBead-activated PBMC, MACSiBead-activated PBMC cocultured with efavirenz or the SDN counterpart (10 µM), PBMC cultured with efavirenz or its SDN counterpart, (10 µM) and PBMC incubated for 24 h with efavirenz or its SDN counterpart for 24 h prior to activation with MACSiBead particles.

### Preparation and activation of primary monocyte derived macrophage (MDM) from healthy volunteers

PBMC were isolated from healthy volunteer whole blood via density gradient centrifugation using Ficoll-Paque. CD14+ positive cells were isolated from PBMC via magnetic bead based cell separation. CD14+ cells were then cultured for 10 days in Iscove’s Modified Dulbecco’s medium (IMDM) containing human serum (20%) and Macrophage colony stimulating factor (M-CSF, 10 ng/mL). Following differentiation into MDM, cells were incubated in the presence of lipopolysaccharide (LPS, 1 µg/mL), conventional efavirenz (10 µM) or efavirenz SDNs (10 µM) for 24 h. Cell culture supernatant fractions were then harvested for cytokine analysis.

### Measurement of cytokine concentrations in activated PBMC and MDM cultures

Aliquots of culture supernatant fractions (100 µL) were taken for analysis of cytokine secretion. Determination of cytokine concentrations was carried out using multiplex cytokine assays conducted using the Bioplex 200 system (Biorad). IL-2, IL-10 and IFNγ were measured for PBMC stimulation and IL-1β, IL-6, IL-8 and TNFα were measured for MDM stimulation. Briefly, coupled beads (50 µL) were added to every well on a 96 well plate. Plates were washed with wash buffer three times using a vacuum manifold prior to cell culture supernatant fractions (50 µL) being added to the plate alongside multiplexed standard curves for relevant cytokines. Samples were incubated on a plate shaker at room temperature for 30 min. Plates were then washed three times prior to the addition of detection antibodies (25 µL) and then incubated on a plate shaker at room temperature for 30 min. Plates were again washed three times prior to the addition of streptavadin-PE antibodies (50 µL) and incubation on a plate shaker for 10 min. Plates were then washed for a final three times and assay buffer (125 µL) added to wells. Plates were then analysed on a Bioplex 200 analyser using recommended gating settings.

### Analysis of activation markers in CD4 and CD8 T cells

Prior to analysis of activation marker expression by flow cytometry MACSiBeads were removed from cell cultures by resuspending PBMC in buffer (PBS, pH7.2; 0.5% human serum albumin; 2 mM EDTA) to a density of 5 × 10^6^/mL and placing the tube in a magnetic field (MACSiMAG separator) for 5 min. The supernatant fraction containing the cells was carefully removed and placed in a fresh tube. The tube was removed from magnetic field, buffer added and the process repeated to maximise recovery of cells. PBMC samples were then stained with either CD4-FITC or CD8-FITC conjugated antibodies (1:11) in buffer for 30 min to enable gating of CD4+ and CD8+ T cells along with a combination of antibodies against either CD25-PE, CD44-APC, CD69-APC or CD95-APC. Samples were then washed three times in buffer before analysis on a BD FACSCantoII flow cytometer. The PBMC population was gated using linear forward and side scatter.

### Analysis of cell proliferation in PBMC treated with PHA by incorporation of ^3^H-thymidine

Cell density of PBMC was adjusted to 2.5 × 10^6^ cells per mL and plated at 100 µL per well (25,000 cells per well) in a 96 well round bottomed plate. 50 µL of phytohaemagglutinin (PHA) (20 µg/mL) was then added per well. 50 µL of either medium or medium containing drug (efavirenz or efavirenz SDNs, 10 µM) was added to the wells in triplicate. Plates were then cultured for 48 h (37 °C; 5% CO_2_), the final 16 h with 1 µCi [^3^H]-thymidine per well. Cell were then harvested onto a filtermat using a tomtec harvester 96 and sealed in a sample bag with melt on scint. Incorporated radioactivity was counted on a Perkin-Elmer MicroBeta detector.

### Analysis of phagocytosis in primary monocyte derived macrophages

CD14+ cells were isolated from PBMC samples by magnetic bead separation and incubated in IMDM media containing M-CSF (10 ng/mL) for 12 days replacing the media every 3 days to differentiate into monocyte derived macrophages (MDM). Following differentiation, MDM were treated with efavirenz or the efavirenz SDNs (10 µM) for 24 h. Following incubation, determination of phagocytic capacity was achieved using pHrodo reagent (Molecular probes, UK). Briefly, MDM were harvested and plated out in a black 96 well plate at 100,000 cells per well. pHrodo™ BioParticles^®^ were prepared by resuspending 2 mg of lyophilized product in 2 mL of uptake buffer (Hanks Balanced Salt Solution, 20 mM HEPES, pH 7.4) and briefly vortexed to completely resuspend the particles. Cytochalasin B (10 µM) was used as a positive control for inhibition of phagocytosis. Culture media was removed from each well and replaced with the pHrodo bioparticle solution (100 µL). The plate was covered and transferred to an incubator at 37 °C without CO_2_ to prevent artificial acidification of the uptake buffer thereby minimising background signal. Plates were read using a plate reader with an excitation of 550 nM and emission of 600 nM.

### Accumulation of radiolabelled efavirenz solid drug nanoparticles in monocyte derived macrophages

Monocyte derived macrophages were generated as described above and treated with Dynasore (100 µM), indomethacin (100 µM) or cytochalasin B (5 µM) for 24 h prior to the addition of radiolabelled efavirenz SDNs (0.3 µCi, 10 µM) for 1 h. Following incubation cells were centrifuged (9000 rpm, 1 min) and an aliquot of supernatant fraction (100 µL, extracellular sample) taken and placed in a scintillation vial.

### Measurement of efavirenz solid drug nanoparticle effects on cytotoxic activity of NK cells by label-free RT-CES system

Effector cells (NK92) were prepared at 1 × 10^6^ cells/mL and treated with test nanoparticles for 24 h. Target cell (HepG2) density was adjusted to 0.5 × 10^6^ cells/mL. Media (50 µL RPMI-1640) was added to all wells and plate attached to RT-CES, starting the appropriate program. Following background measurement, HepG2 (50 µL) were added per well of the RT-CES plate and data acquisition started. HepG2 cells were left in culture for approximately 16–20 h prior to the addition of NK92 cells. On the 2nd day, NK92 cell viability was determined and cells readjusted to a density of 25. × 10^6^ viable cells/mL resulting in an effector to target (E:T) ratio of 5:1. RT-CES program was paused for the addition of NK92 cells then returned to incubator to resume data acquisition for a further 24 h. On day 3, data acquisition was stopped and the data analysed by assessing the area under the curve (AUC) for each sample.

### Statistical analysis

Distribution of the data was assessed using a Shapiro–Wilk test. For comparisons between datasets either an unpaired t test or a Mann–Whitney test was used for normally and non-normally distributed data respectively. Stats Direct software (version 3.0.171) was used for data analysis and a P value < 0.05 was considered statistically significant.

## Results

### Characterisation of efavirenz solid drug nanoparticles

A 1 mg/mL (with respect to EFV content) dispersion of efavirenz SDN was made in deionised water, phosphate buffered saline (PBS) or NaCl (10 mM). Hydrodynamic diameter (Z-average) and zeta potential were assessed in each of these dispersions to determine their impact on physical-chemical characteristics (PCC). There was no significant difference in size for SDNs dispersed in deionised water (264 ± 5 nm) compared to SDN dispersed in PBS (300 ± 16 nm) or NaCl (289 ± 12 nm; Additional file 2: Figure S1a). Additional file 2: Figure S1b shows polydispersity indices (PDI) for SDNs dispersed in deionised water, PBS and NaCl. Efavirenz SDNs dispersed in PBS (0.402 ± 0.055; P < 0.001) and NaCl (0.377 ± 0.064; P < 0.001) had a significantly greater PDI than those in deionised water (0.278 ± 0.035). Efavirenz SDN zeta potential were also assessed at native pH of the dispersion (pH 7.89) and titrated to neutral pH (7.09). The zeta potential was nearly neutral in both cases.

### Assessment of possible endotoxin or microbial contamination

Using a turbidimetric LAL assay the concentration of endotoxin was assessed in dispersed SDNs as well as an aqueous solution of EFV. The mean concentration of endotoxin in the aqueous solution of EFV and the EFV SDN dispersion was found to < 0.001 and 0.0306 EU/mL respectively. This was confirmed by gel-clot LAL assay. In order to ensure there was no inhibition of the LAL assay by a known concentration of endotoxin (0.05 EU/mL) was spiked into sample materials prior to turbidimetric LAL analysis with an acceptable range of recovery between 50 and 200%. Recovery of endotoxin within aqueous EFV and EFV SDN was 179 and 180% respectively and therefore within acceptable parameters.

Additionally, H460 cells were treated with 1 mg/mL of either efavirenz solution or efavirenz SDN and passaged every 48 h for 18 passages. Samples were taken at every passage and then samples were analysed by PCR for mycoplasma contamination. No mycoplasma was detected after treatment with sample materials.

Sample materials were spread on LB agar plates at a concentration of 4 and 40 µg/mL and incubated for 48 h; images of the culture plates are shown in Additional file 3: Figure S2. A culture of *E. coli* was also plated out as a positive control and Additional file 3: Figure S2a shows growth after 48 h whilst a negative control of LAL reagent water shows no growth (Additional file 3: Figure S2b) after the same period of time. No growth was observed for either aqueous EFV or EFV SDN at either 4 or 40 µg/mL after 48 h.

### Impact of efavirenz solid drug nanoparticles on platelet aggregation

**Fig. 1:**
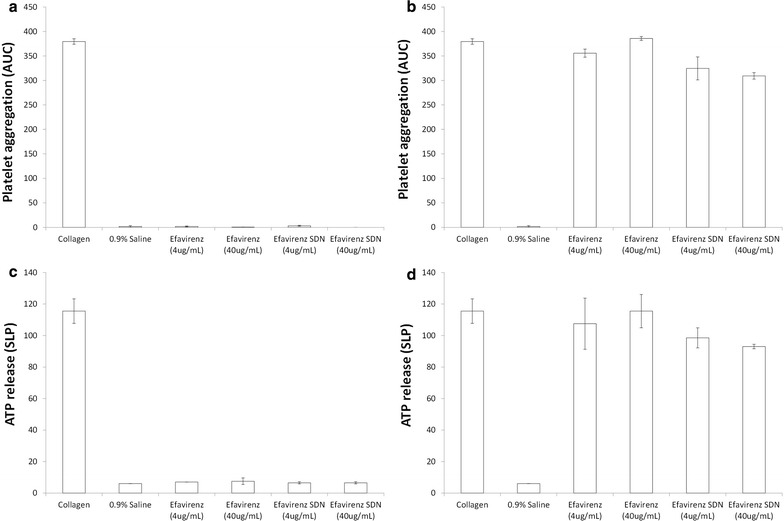
Response of primary human platelets to efavirenz and efavirenz solid drug nanoparticles. Platelet aggregation was assessed in response to materials alone (**a**) and that induced by collagen (**b**). Platelet activation was assessed by ATP release in non-induced (**c**) and collagen induced (**d**) platelets. Data expressed as mean ± standard deviation

Efavirenz SDNs were tested for their impact on platelet aggregation and activation at 4 and 40 µg/mL. Saline (0.9%) was used as a negative control for platelet activation and collagen (1 µg/mL) used as a positive control. Platelet aggregation was defined as the area under the curve (AUC) for the turbidimetric assessment and activation by the slope of the curve produced for ATP release. Collagen resulted in a 300-fold greater AUC than that generated by saline solution. Neither the efavirenz solution, nor SDNs, caused aggregation of platelets at either 4 or 40 µg/mL (Fig. 1a). Platelet aggregation was induced by collagen and samples treated with aqueous efavirenz solution or efavirenz SDN. No significant impact on collagen induced platelet aggregation was observed (Fig. 1b). These observations were further confirmed by ATP release from platelets. Tested materials did not cause significant ATP release from platelets nor did they inhibit ATP release from platelets activated by collagen (Fig. 1c, d, respectively).

### Impact of efavirenz solid drug nanoparticles on plasma coagulation pathways

**Fig. 2:**
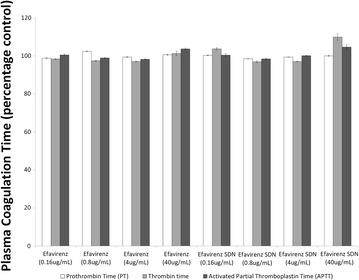
Impact of sample materials on plasma coagulation times. Plasma coagulation times assessed for the three main coagulation pathways in healthy volunteer blood treated with efavirenz aqueous solution or efavirenz SDN. Data are shown as the mean ± standard deviation

The impact of efavirenz solution and efavirenz SDNs on the three main pathways of plasma coagulation (intrinsic, extrinsic and common pathways) was assessed by measuring activated partial thromboplastin time (APTT), prothrombin time (PT) and thrombin time (TT), respectively. A prolongation of plasma coagulation time is associated with interference with coagulation cascades. Plasma coagulation times for the three pathways studied following treatment is summarised in Fig. 2. No marked difference in plasma coagulation time for samples treated with either efavirenz aqueous solution or efavirenz SDNs.

### Assessment of potential haemolysis caused by efavirenz solid drug nanoparticles

**Fig. 3:**
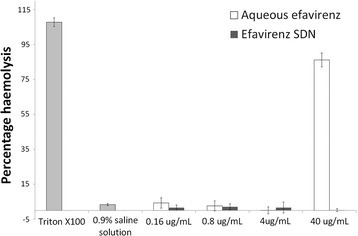
Extent of haemolysis in healthy volunteer blood treated with efavirenz aqueous solution and efavirenz SDN. Healthy volunteer blood was treated with a range of concentrations of test materials for 3 h at 37 °C. Percent haemolysis was determined by assessing the amount of free haemoglobin present in samples post incubation

Haemolysis was assessed by determining the amount of free haemoglobin in samples treated with either aqueous efavirenz or efavirenz SDNs. Efavirenz aqueous solution and SDNs were tested at concentrations of 0.16, 0.8, 4 and 40 µg/mL efavirenz. Materials were also tested in the absence of blood to ensure no interference with the assay. A percentage haemolysis between 5 and 25% has previously been accepted as not of any direct concern [46–49]. Figure 3 summarises the percentage haemolysis caused by treatment of blood with efavirenz solution or efavirenz SDNs. Treatment of blood with 0.9% saline solution resulted in 3.3% haemolysis. Haemolysis caused by 0.16, 0.8, 4 and 40 µg/mL efavirenz solution was 4.29, 2.61, 0.04 and 86.2%, respectively. Percentage haemolysis caused by efavirenz SDNs at 0.16, 0.8, 4 and 40 µg/mL was 1.6, 2.08, 1.63 and 0.17%, respectively.

### Determination of possible complement activation

**Fig. 4:**
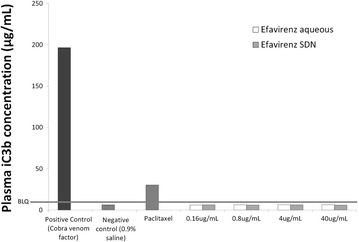
Concentration of iC3b in plasma treated with sample materials. Pooled plasma (N = 3) was treated with efavirenz aqueous solution or efavirenz SDN. Concentration of iC3b was determined by ELISA. *BLQ* below limit of quantification of assay

The concentration of iC3b in plasma treated with efavirenz aqueous solution or efavirenz SDNs (summarised in Fig. 4) was determined as a possible measure of complement activation by either the classical or alternative pathway. Cobra venom factor (positive control) yielded a very strong increase in the concentration of iC3b (196.8 µg/mL) whereas 0.9% saline (negative control) yielded only 6.3 µg/mL of iC3b, which was below the limit of quantification of the assay. Similarly for all tested materials, no activation of complement occurred, as evidenced by increased concentrations of iC3b, since all values were below the limit of quantification. Paclitaxel was included as a clinically relevant positive control with known activation of complement [50] and was shown to result in an increase in iC3b concentrations (30 µg/mL).

### Impact of efavirenz solid drug nanoparticles on T cell function and phenotype

**Fig. 5:**
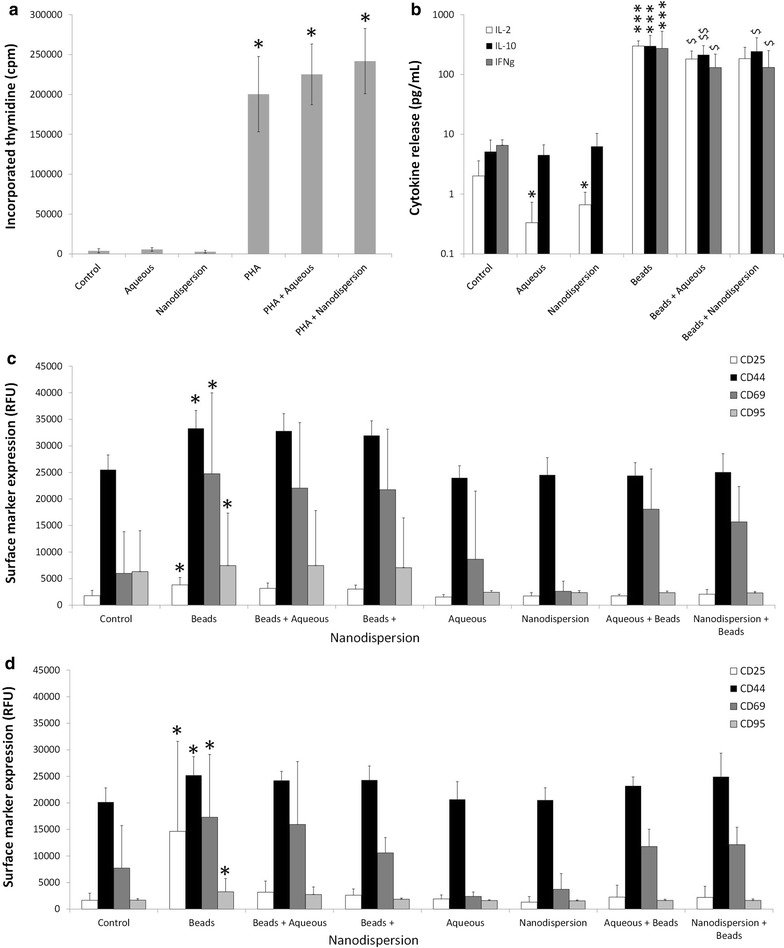
Characterisation of the potential interaction between solid drug nanoparticles and lymphocyte function. PBMC were treated with either efavirenz aqueous solution or efavirenz s for 24 h before their proliferative capacity (**a**), cytokine secretion (**b**) and surface marker expression in CD4+ (**c**) and CD8+ (**d**) cells were determined. Data expressed as mean ± standard deviation (N = 5); *P < 0.05, **P < 0.01, ***P < 0.001 as compared to untreated control whereas ^$^P < 0.05, ^$$^P < 0.01, ^$$$^P<0.001 compared to positive control

The proliferation of lymphocytes in response to PHA and/or sample materials was assessed using ^3^H-thymidine incorporation. There was no significant difference in ^3^H-thymidine incorporation between untreated, control cells and cells treated with either efavirenz solution or SDNs (Fig. 5a). Treatment with PHA caused proliferation and thereby significantly increased ^3^H-thymidine incorporation in PHA treated cells compared to untreated cells (51-fold increase, P = 0.02). A similar fold increase was observed for cells treated with PHA and efavirenz aqueous solution (57-fold increase, P = 0.01) and cells treated with PHA and efavirenz SDNs (61-fold increase, P = 0.01). No significant difference was observed in the proliferation of cells treated with PHA with efavirenz aqueous solution (P = 0.16) and PHA with efavirenz SDNs (P = 0.18).

Secretion of IL-2, IL-10 and IFNγ was assessed in PBMC samples treated with sample materials (Fig. 5b) for 24 h. Anti-CD2/CD3/CD28 beads were used as a positive control for stimulation of T cells and were shown to significantly increase the secretion of IL-2 (148-fold greater, P = 0.0002), IL-10 (58-fold greater, P = 0.0006) and IFNγ (42-fold greater, P = 0.0002). Secretion of IL-10 from cells treated with efavirenz aqueous solution or efavirenz SDNs was not significantly different from untreated cells. However, IL-2 secretion was significantly lower from cells treated with efavirenz aqueous solution (sixfold lower, P = 0.015) and efavirenz SDNs (threefold lower, P = 0.0008). IFNγ was similarly affected but concentrations were reduced to below the limit of detection rendering statistical comparison impossible. IL-2 secretion from cells stimulated with beads and treated with efavirenz aqueous solution (1.6-fold lower, P = 0.038) and efavirenz SDN (1.6-fold lower, P = 0.09) was significantly lower than that of cells stimulated solely with beads but there was no significant difference between the two treatments. A similar effect was observed for IL-10 (efavirenz aqueous solution, 1.4-fold lower, P = 0.0035; efavirenz SDNs, 1.2-fold lower, P = 0.04) and IFNγ (efavirenz aqueous solution, twofold lower, P = 0.039; efavirenz SDNs, twofold lower, P = 0.037). However, there was again no significant difference was observed between solution and SDNs.

Surface markers of T cell activation were measured in CD4+ and CD8+ cells and data are summarised in Fig. 5c, d, respectively. On CD4+ cells, expression of CD25 (twofold greater, P = 0.045), CD44 (1.3-fold greater, P = 0.015), CD69 (fourfold greater, P = 0.03) and CD95 (1.2-fold greater, P = 0.045) was higher than in unstimulated cells. Similar effects were seen in CD8 + cells with respect to CD25 (eightfold greater, P = 0.045), CD44 (1.25-fold greater, P = 0.05), CD69 (2.2-fold greater, P = 0.01) and CD95 (1.9-fold greater, P = 0.04) expression. Incubation of cells with either efavirenz aqueous solution or efavirenz SDNs did not significantly affect the expression of surface markers of activation.

### Impact of solid drug nanoparticles on monocyte derived macrophage function

**Fig. 6:**
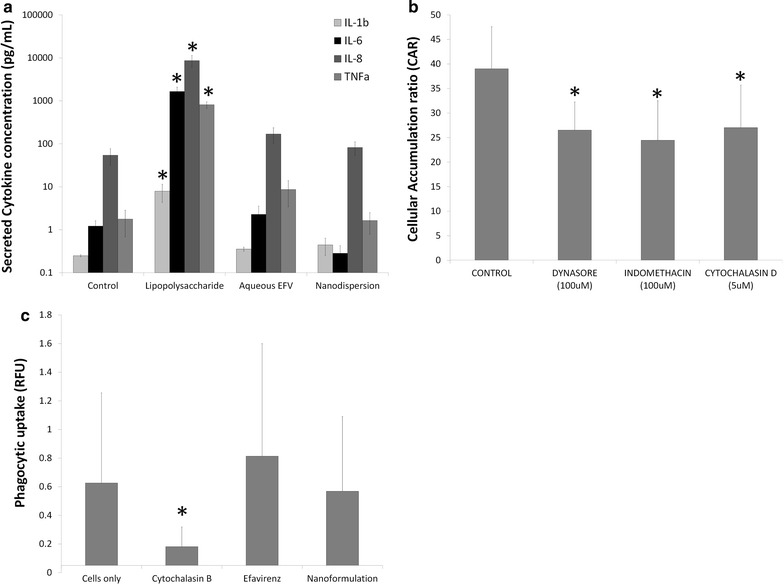
Characterisation of the potential impact of efavirenz solid drug nanoparticles on monocyte derived macrophage function. MDM were treated with either efavirenz aqueous solution or efavirenz s for 24 h before their cytokine secretion (**a**) was assessed. LPS included as a positive control. Uptake of efavirenz s was also assessed (**b**) in addition to the phagocytic capacity of MDM treated with sample materials. Data expressed as mean ± standard deviation (N = 5); *P < 0.05, **P < 0.01, ***P<0.001 as compared to untreated control

Monocyte derived macrophages (MDM) were treated with LPS, efavirenz aqueous solution or efavirenz SDNs for 24 h (Fig. 6a). Following incubation, aliquots of cell culture supernatant fraction were taken and analysed for concentrations of IL-1β, IL-6, IL-8 and TNFα. LPS treatment resulted in greater secretion of IL-1β (31-fold greater, P = 0.03), IL-6 (1360-fold greater, P = 0.03), IL-8 (158-fold greater, P = 0.03) and TNFα (458-fold greater, P = 0.03). Neither efavirenz aqueous solution nor efavirenz SDNs significantly affected cytokine secretion from MDM.

The accumulation of efavirenz SDNs in MDM was also assessed incorporating inhibitors of clathrin mediated endocytosis (Dynasore), caveolae mediated endocytosis (indomethacin) and phagocytosis (cytochalasin B) (Fig. 6b). All inhibitors reduced the accumulation of efavirenz SDNs (Dynasore, 1.47-fold lower accumulation, P = 0.047; Indomethacin, 1.59-fold lower accumulation, P = 0.047; cytochalasin B, 1.44-fold reduction, P = 0.048).

As phagocytosis appears to be involved in the uptake of efavirenz SDNs their impact on the phagocytosis of fluorescent *E. coli* bioparticles by MDM was also assessed to ensure no interference with function (Fig. 6c). Cytochalasin B was included as a positive control and showed a 3.4-fold lower (P = 0.04) accumulation of bioparticles in MDM. Uptake of fluorescent bioparticles by MDM was not significantly affected by either efavirenz aqueous solution or efavirenz SDNs (Fig. 6c).

### Impact of efavirenz and efavirenz solid drug nanoparticles on NK cell cytotoxicity

**Fig. 7:**
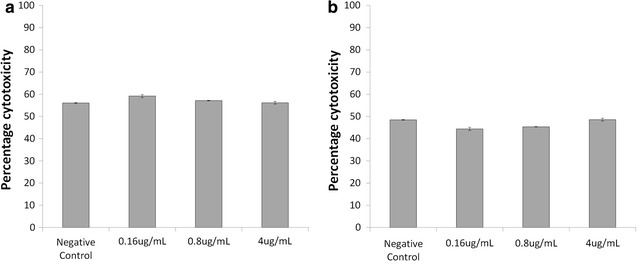
Cytotoxicity of NK92 cells to HepG2 cells following treatment with efavirenz and efavirenz solid drug nanoparticles. NK92 cells were treated with either efavirenz aqueous solution (**a**) or efavirenz s (**b**) for 24 h before their cytotoxic capability was assessed

NK cell cytotoxicity was assessed using NK92 cells as effector cells and HepG2 as target cells and measuring cell viability via electrical impedance generated by HepG2 attachment to RT-CES plates. Treatment of HepG2 cells with NK92 cells resulted in a 52% lower viability compared to HepG2 cells grown in monoculture (Fig. 7). Treatment of NK92 cells with efavirenz aqueous solution for 24 h resulted in no significant change in their cytotoxic capability (Fig. 7a). Similarly, treatment of NK92 cells with efavirenz SDNs did not significantly affect their cytotoxic capability (Fig. 7b).

## Discussion

Understanding the interaction with immunological and haematological systems is vital to the preclinical evaluation of novel nanoparticle formulations for the treatment of disease. To date, only one study has investigated the impact of SDNs on immune function [51]. In order to determine whether SDNs composed of another drug behaved differently, a series of immune-activation criteria were conducted to assess whether the efavirenz nanoparticles trigger or interfere with immune responses. Throughout the experimental assessment, all incubations were conducted with equivalent drug concentrations of efavirenz between nanoparticle and aqueous solutions. Therefore, differences are due only to the nanoparticulate nature of the formulation.

Microbial contamination that may occur during the manufacturing process, particularly the introduction of endotoxin, can provide false positive results in several immunological assays. Prior to analysis of immune interactions, the amount of endotoxin present in both the efavirenz solution and the efavirenz SDNs was determined and found to be almost negligible. Hence, there was no evidence of microbial contamination following culture.

It is currently unclear if SDNs reach the blood, following oral administration, as intact nanoparticles but SDNs are being developed for several parenteral applications. Haemolysis can lead to various pathologies (e.g. anaemia and jaundice) and is a relatively common issue with environmental and medicinal nanoparticles [38, 52]. Therefore, to gain further confidence in SDN safety, we assessed haemolysis after incubation with whole blood from a healthy human volunteer. Aqueous efavirenz caused a significant amount of haemolysis. However, the efavirenz SDNs did not cause haemolysis, which is promising for SDNs being investigated for intravenous delivery. No impact on blood coagulation time in response to coagulation inducers was evident at the concentrations tested. Platelet aggregation was similarly unaffected by efavirenz SDNs and the test materials did not interfere with platelet activation induced by collagen. Complement activation has been reported for nanomaterials previously [50] so measurement of iC3b was conducted (the common factor for all three complement pathways) in healthy volunteer plasma. No activation of iC3b was observed following treatment suggesting that efavirenz SDNs do not activate complement via any of the main pathways.

No differences in phagocytic capacity were observed between MDM treated with SDNs or aqueous solution. Stimulation of MDM was also assessed using a panel of cytokines previously shown to be associated with macrophage activation. Neither the aqueous solution nor SDNs significantly stimulated cytokine secretion from MDM. Additionally, no difference in proliferation of PBMC from healthy volunteers was observed for SDNs or the aqueous solution, and neither interfered with proliferation in response to the known mitogen, PHA. The secretion of cytokines from PBMCs (IL-2, IL-10 and IFNγ) was also similar for SDNs and aqueous solution, as was the impact on cytokine release in response to anti-CD3/anti-CD28 beads. IL-2, IL-10 and IFNγ were chosen because of their secretion from T cells following T cell receptor activation [53–55]. Anti CD3/anti-CD28 beads bind and activate CD3 (T-cell receptor) and CD28 (co-stimulatory receptor) and are an established tool for inducing T-cell activation [56–58]. Finally, the expression of cell surface receptors associated with T-cell activation [59–62] was monitored in response to incubation with the efavirenz SDNs or an efavirenz aqueous solution. Again, no differences in expression in either CD4+ or CD8+ T cells between SDNs and an aqueous solution and no differences in the response to anti-CD3/antiCD28 beads were observed. NK92 cells were used as a model of primary NK cells to assess potential immunosuppression by efavirenz SDNs and no impact on NK92 cytotoxicity was observed. Therefore, in this ex vivo analysis, no immunological safety concerns were uncovered using the employed assays. Contrary to other reported nanoparticles, the SDNs presented here do not appear to interact with the immune system in the same way. Taken collectively with the previous observations with lopinavir SDNs, this particular nano-specific interaction does not appear to be a concern with SDN formulations, which bodes well for parenterally administered materials of this type. However, it should be noted that since SDNs are manufactured from the drug itself, one cannot rule out that their formation will not confer augmentation of molecule-specific interactions.

In summary, a previously pharmacologically optimised SDN formulation of efavirenz with improved bioavailability, in vitro cellular distribution and antiretroviral activity [43], did not interfere with components of the immune system in the studied assays, and exhibited favourable blood-contact properties. The emulsion-templated freeze drying platform may have broad application for optimising drugs with solubility/bioavailability issues. All excipients used are on the FDA CDER list and the resultant SDNs are physically similar to currently licensed SDN products.

## Conclusions

The pharmacologically optimised efavirenz SDNs presented here have shown to be immunologically and haematologically inert in the in vitro/ex vivo preclinical assessment. The methodologies utilised can now be used to assess future nanomaterials as part of a preclinical assessment, to de-risk translation into clinical evaluation.

## Additional files

**Additional file 1: Table S1.** Guide to settings and reagent requirements for assessment of plasma coagulation times of human plasma in response to treatment with nanoparticles.

**Additional file 2: Figure S1.** Physical-chemical characterisation of efavirenz solid drug nanoparticles. SDNs were dispersed in deionised water, PBS or NaCl (10mM) at a concentration of 1mg/mL. Sample hydrodynamic size (a) and polydispersity index (b) were assessed using dynamic light scattering (DLS) analysis. Zeta potential (c) was also assessed at two different pH.

**Additional file 3: Figure S2.** Detection of possible microbial contamination in sample materials via growth on LB agar plates. Sample materials were spread on LB agar plates and incubated for 48 hours in a humidified incubator. *E. coli* (a) was used as a positive control for microbial growth while LAL reagent water (b) was used as a negative control. Aqueous efavirenz was tested at 4µg/mL (c) and 40µg/mL (d) as well as efavirenz SDN (e, 4µg/mL and f, 40µg/mL).

## Abbreviations

APC: allophycocyanin
API: active pharmaceutical ingredient
aPTT: activated partial thromboplastin time
ATP: adenosine triphosphate
AUC: area under the curve
CD-: cluster of differentiation-
CMH: cyanomethaemoglobin
CV: coefficient of variation
DIC: disseminated intravascular coagulopathy
DMSO: dimethyl sulphoxide
DPBS: Dulbecco’s phosphate buffered saline
EFV: efavirenz
ELISA: enzyme linked immunosorbant assay
EU: endotoxin units
FBS: foetal bovine serum
FITC: fluorescein isothiocyanate
HEPES: 4-(2-hydroxyethyl)piperazine-1-ethanesulfonic acid, *N*-(2-hydroxyethyl)piperazine-*N*′-(2-ethanesulfonic acid)
IEC: inhibition/enhancement controls
IFNγ: interferon gamma
IL-: interleukin-
IMDM: Iscove’s Modified Dulbecco’s medium
LAL: limulus amoebocyte lysate
LB: Luria broth
LPS: lipopolysaccharide
M-CSF: macrophage colony stimulating factor
MDM: monocyte derived macrophages
NK: natural killer
PAMAM: polyaminoamine
PBMC: peripheral blood mononuclear cells
PCC: physical–chemical characteristics
PCR: polymerase chain reaction
PDFT: percent difference from theoretical
Pdi: polydispersity index
PE: phycoerythrin
PFC: perfluorocarbon
PFH: plasma free haemoglobin
PHA: phytohaemagglutinin
PLGA: poly(lactic-*co*-glycolic) acid
PPP: Platelet Poor Plasma
PRP: platelet rich plasma
PT: prothrombin time
PVA: poly(vinyl alcohol)
rpm: revolutions per minute
RT-CES: real time cell electronic sensing
SDN: solid drug nanoparticle
SDN: standard deviation
TBH: total blood haemoglobin
Th1: T helper 1
Th2: T helper 2
TNFα: tumour necrosis factor α
TPGS: d-α-tocopherol polyethylene glycol succinate
TT: thrombin time
