# Supplementary figures and images for: Assessment of interactions of efavirenz solid drug nanoparticles with human immunological and haematological systems
Source: J Nanobiotechnology. 2018 Mar 15;16:22. doi: 10.1186/s12951-018-0349-y (PMC5853089; doi:10.1186/s12951-018-0349-y)

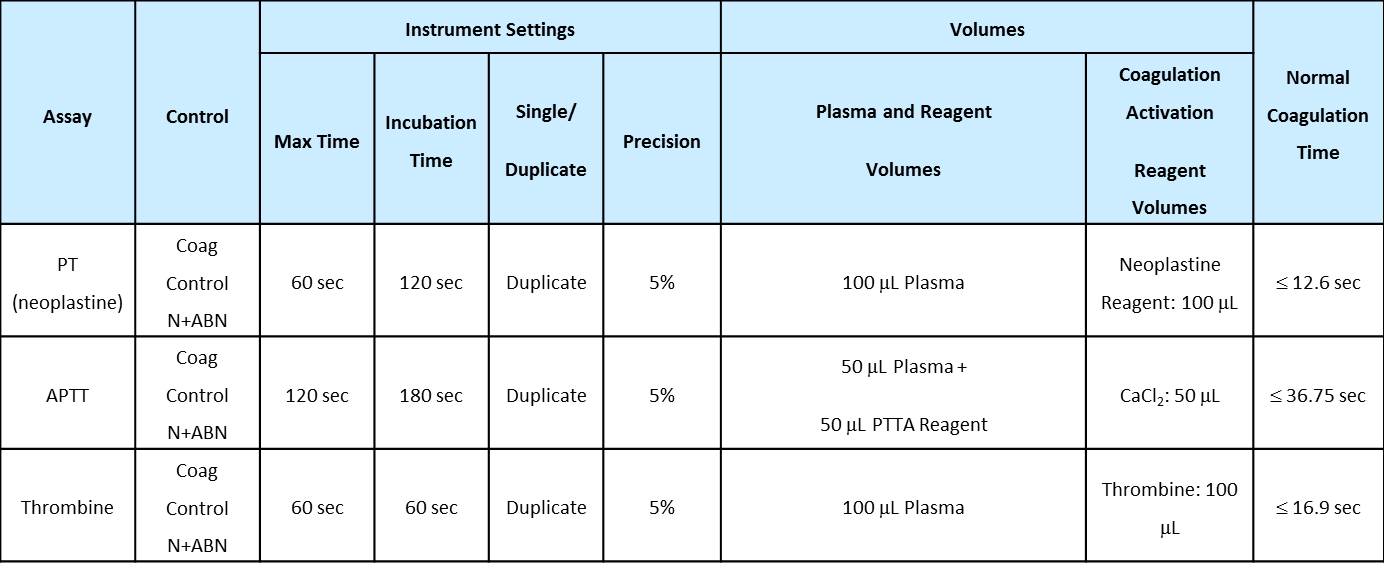

Supplement: Supplementary file 1 — Additional file 1: Table S1. Guide to settings and reagent requirements for assessment of plasma coagulation times of human plasma in response to treatment with nanoparticles. [file 12951_2018_349_MOESM1_ESM.jpg]

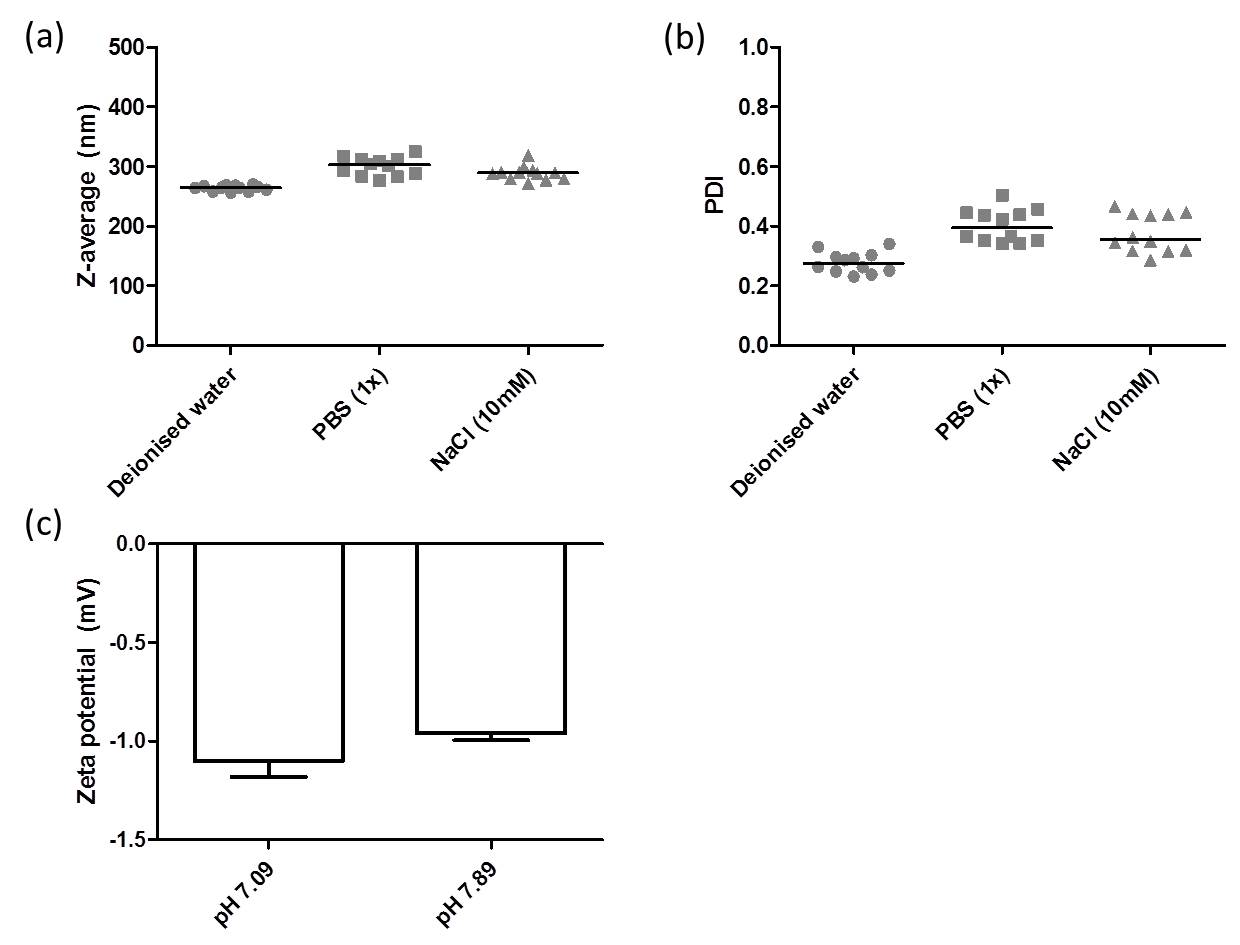

Supplement: Supplementary file 2 — Additional file 2: Figure S1. Physical-chemical characterisation of efavirenz solid drug nanoparticles. SDNs were dispersed in deionised water, PBS or NaCl (10mM) at a concentration of 1mg/mL. Sample hydrodynamic size (a) and polydispersity index (b) were assessed using dynamic light scattering (DLS) analysis. Zeta potential (c) was also assessed at two different pH. [file 12951_2018_349_MOESM2_ESM.jpg]

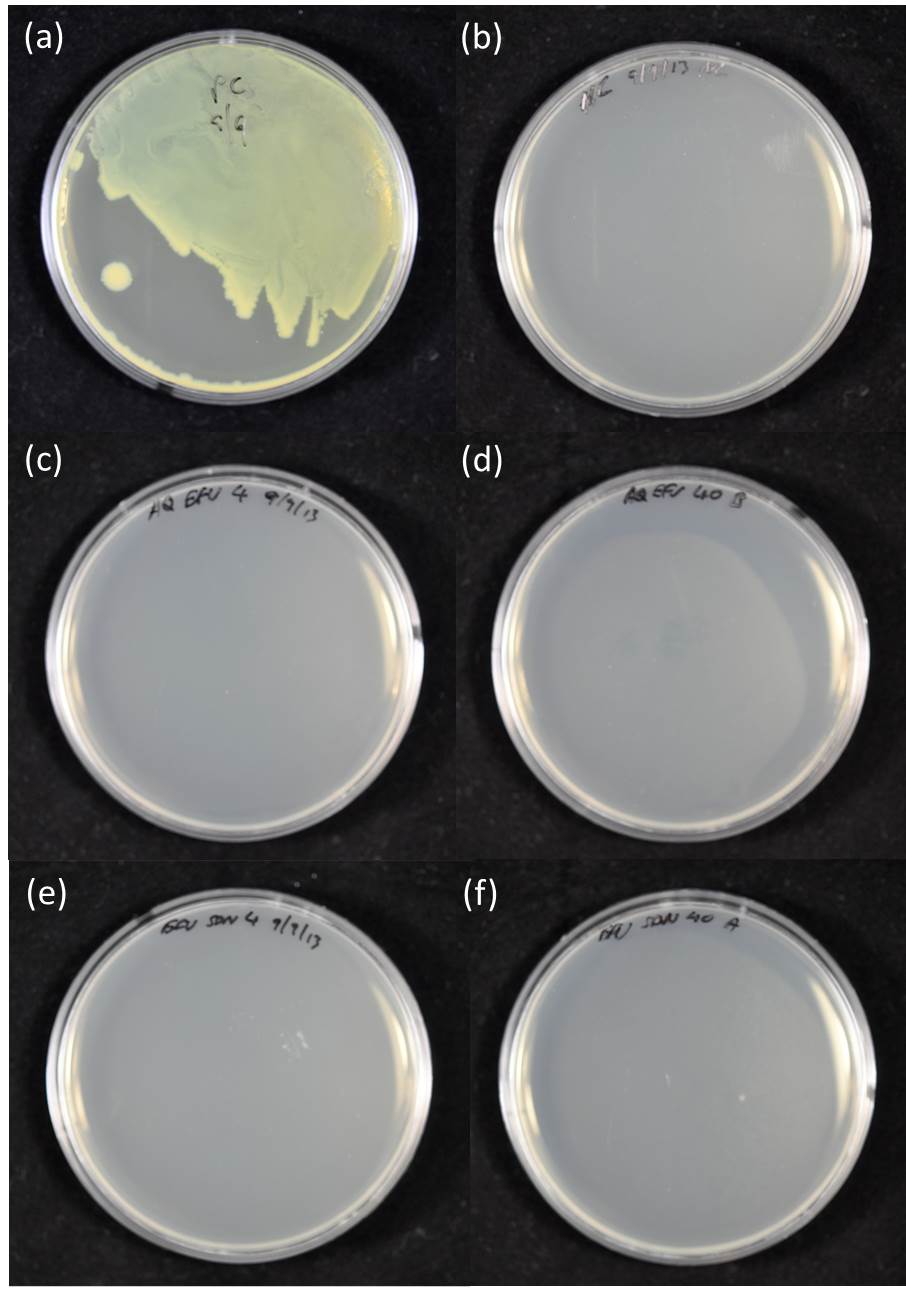

Supplement: Supplementary file 3 — Additional file 3: Figure S2. Detection of possible microbial contamination in sample materials via growth on LB agar plates. Sample materials were spread on LB agar plates and incubated for 48 hours in a humidified incubator. E. coli (a) was used as a positive control for microbial growth while LAL reagent water (b) was used as a negative control. Aqueous efavirenz was tested at 4µg/mL (c) and 40µg/mL (d) as well as efavirenz SDN (e, 4µg/mL and f, 40µg/mL). [file 12951_2018_349_MOESM3_ESM.jpg]
